# The Holloway Sanatorium

**Published:** 1895-12-07

**Authors:** 


### The Hospital

AN INSTITUTIONAL JOURNAL OF

The Medical Sciences and Hospital Administration,

WITH

AN EXTRA NURSING SUPPLEMENT.

VOL. XIX.—No. 480.] [DEC. 7, 1895.

### THE HOLLOWAY SANATORIUM.

It is very much to be regretted that certain portions of the Press lend themselves far too readily to attempts to inflame the public mind on the subject of the wrongs of lunatics. The insane are the most pitiable of all classes of the sick, and at the same time the most difficult to treat. Society is easily excited from time to time by sensational stories of "madhouses," but society will have nothing to do with lunatics. Insanity in a family is still looked on as a blot, as something to be hidden and stowed away, something that is not to come between the wind and our gentility. The old prejudice against the insane, the creepy feeling of horror and dread of everything connected with insanity, is nearly as strong as ever. The shuddering and mysterious horror with which society regards the institution for the insane is really the reverse side of the old superstitious dread of the insane themselves. Asylums are, no doubt, an evil in the same sense as hospitals are, but they are just as necessary, it might be argued even more so. That individual cases of error and mismanagement will occur from time to time in asylums, as in all other public institutions, is unhappily inevitable so long as we can find no better agents than men and women whereby to manage them. Most of our readers are by this time familiar with the main facts connected with the case of poor Mr. Thomas Weir, who died at the Holloway Sanatorium. Before discussing the lessons that this case teaches, we would like to briefly say what it does not teach. It does not prove the depravity of the owners of proprietary asylums, inasmuch as the Holloway Sanatorium is not a private asylum, and whatever criticism may be offered upon its management nobody can pretend to believe that it is run for the profits of anyone. The stock complaint against private asylums amounts to this, that sane people are retained in them for profit. How is this consistent with statements that patients are "foully done to death" in these establishments, and so forth? However logically weak the position of asylum proprietors may be, they are surely not to be charged with acting on motives inconsistent with all human reasoning and mutually contradictory of each other. In a long article on the subject in the *Nottingham Argus* of November 23rd investigation is called for in the case of Thomas Weir, though it is really hard to see what more can be elicited than has already been made public in the many inquiries which have been held. In this article it is conveyed that poor Weir was placed in restraint merely to prevent his escape, and in a leaflet which Mr. J. Weir, the father of the deceased, has addressed to the readers of the paper this passage occurs: "Picture to yourselves a Beloved Son or Daughter consigned to the care of such persons as control this institution. The case of Miss Lanchester proves how easy the process is, and then upon any show of excitement or desire to escape, Mechanical Restraint comes into play, and the Poor Victim's doom is sealed." Every humane person must be sorry for poor Mr. Weir, senior, but our sympathies must not altogether carry us away. The case of Miss Lanchester surely shows how easy it is to get out of an asylum, even a real private asylum. It has been evidently seized hold of in this connection, because being much talked of just now it formed a convenient means of appealing to popular prejudice. In the worst of the old times of mechanical restraint it did not always or often seal the victim's doom. Nay, as every one familiar with the history of the subject knows, many patients lived for years in continuous restraint, and it could not have been applied as a means of killing patients, since its use was at one time almost universal.

Now what do we learn from this unhappy case? Primarily, the old lesson—and we regret that it should have been necessary to teach it again in England—that mechanical restraint in any form is fraught with danger and is singularly open to misuse. In many asylums restraint is never employed and is never missed. Many experienced superintendents, while unwilling to commit themselves to an absolute statement that restraint should never be made use of, find that they can in practice entirely dispense with it. If we could think that restraint was really beneficial to the insane, we might forget our detestation of it; but even then we would retain the feeling that it is a dangerous and a desperate resource, considering its obvious tendency to degrade both patients and attendants and to encourage negligence among the latter. But there is another lesson. Since restraint can be done without, it certainly never should be employed without the closest medical supervision. It should be dealt with as we would deal with some potent drug—as an agent to be watched with the most minute care—and the employment of restraint should no more become a matter of routine than the administration of hyoscine.

In cases of acute mania there is a great tendency to death by exhaustion, and the coroner's jury who inquired into poor Weir's case no doubt found a just verdict when they attributed his death to that cause. It is to be regretted that they had it in their power to add a rider to the effect that in their opinion sufficient supervision was not exercised, and that restraint was excessive and too long continued, even though it were maintained, as it very plausibly might be, that these conditions did not contribute to the lamentable termination.

Those who have attacked the whole management of the institution in a very unwarrantable manner, and, it may be feared, under the influence of motives not impersonal, have made no allowance for the fact that at this crisis the medical staff was weakened by sickness. In using such a weapon as mechanical restraint one needs to have the aid of a staff thoroughly capable both physically and otherwise. However, no one can be wise at all times, and it is always the unexpected that happens, at least in asylums. The fury with which the staff of the Holloway Sanatorium have been attacked has excited the sympathy of many who have little sympathy with "restraint," but do not think that errors of judgment should be treated as crimes or that any class of man should have motives attributed to them at once of the vilest and most incredible nature.

